# Integrated pan-cancer analysis of CSMD2 as a potential prognostic, diagnostic, and immune biomarker

**DOI:** 10.3389/fgene.2022.918486

**Published:** 2022-08-17

**Authors:** Huiyun Zhang, Taobi Huang, Xiangqing Ren, Xidong Fang, Xia Chen, Hui Wei, Weiming Sun, Yuping Wang

**Affiliations:** ^1^ The First Clinical Medical College, Lanzhou University, Lanzhou, China; ^2^ Department of Gastroenterology, The First Hospital of Lanzhou University, Lanzhou, China; ^3^ Key Laboratory for Gastrointestinal Diseases of Gansu Province, The First Hospital of Lanzhou University, Lanzhou, China; ^4^ Department of Endocrinology, The First Hospital of Lanzhou University, Lanzhou, China

**Keywords:** CSMD2, pan-cancer, prognosis, immune, microenvironment, methylation

## Abstract

The protein encoded by CUB and Sushi Multiple Domains 2 (CSMD2) is likely involved in regulating the complement cascade reaction of the immune system. However, current scientific evidence on the comprehensive roles of CSMD2 in pan-cancer is relatively scarce. Therefore, in this study, we explored the transcriptional level of CSMD2 in pan-caner using TCGA, GEO, and International Cancer Genome Consortium databases. Receiver operating characteristic curve analysis was used to investigate the diagnostic efficacy of CSMD2. The Kaplan-Meier Plotter and Oncolnc were used to investigate the correlation between CSMD2 expression and prognosis. Additionally, we analyzed the correlation between epigenetic methylation and CSMD2 expression in various cancers based on UALCAN, as well as, the correlation between CSMD2 and tumor mutational burden (TMB), microsatellite instability (MSI), and tumor neoantigen burden (TNB) in tumors. TIMER2.0 database was employed to investigate the correlation between CSMD2 and immune cells in the tumor microenvironment and immune checkpoints. Based on TISIDB, the correlation between CSMD2 and MHC molecules and immunostimulators was analyzed. Ultimately, we observed with a pan-cancer analysis that CSMD2 was upregulated in most tumors and had moderate to high diagnostic efficiency, and that high expression was closely associated with poor prognosis in patients with tumors. Moreover, hypermethylation of CSMD2 promoter and high levels of m6A methylation regulators were also observed in most cancers. CSMD2 expression was negatively correlated with TMB and MSI in stomach adenocarcinoma (STAD) and stomach and esophageal carcinoma (STES), as well as with tumor mutational burden, microsatellite instability, and TNB in head-neck squamous cell carcinoma (HNSC). In most cancers, CSMD2 might be associated with immune evasion or immunosuppression, as deficient anti-tumor immunity and upregulation of immune checkpoints were also observed in this study. In conclusion, CSMD2 could serve as a promising prognostic, diagnostic and immune biomarker in pan-cancer.

## 1 Introduction

CUB and Sushi Multiple Domains 2 (CSMD2), located on the short arm of human chromosome 1 (1p35.1), is mainly expressed in the brain and gall bladder. Fourteen CUB domains at the N-terminal of CSMD2 are separated by a single complement control protein (CCP) domain, followed by 13 series of CCP domains. CCP is also called short consensus repeats (SCR) or Sushi domain, notably, multiple consecutive CCP domains are common characteristics of many complement inhibitors containing such domains (Rossi et al., 1998; Gialeli et al., 2018). CUB and sushi domains are considered as sites of protein-protein or protein-ligand interactions, indicating that CSMD proteins are either transmembrane receptors or adhesion proteins (Lau and Scholnick, 2003). CSMD2 has been associated with schizophrenia (Håvik et al., 2011). It is downregulated and is associated with the poor prognosis in colorectal cancer (Zhang and Song, 2014). However, current studies on the role of CSMD2 in tumorigenesis and the development of other tumors are lacking. In addition, genetic alterations in CSMD2 have been detected in primary lymphoma of the central nervous system and colorectal cancer and have been found to been associated with prognosis (Vater et al., 2015; Yang *p*.-S. et al., 2018). As a complement system regulator and receptor, its immune role in tumorigenesis and development remains unclear.

Cancer imposes a major burden on human society and was either the first or second leading cause of death before the age of 70 in 112 of 183 countries (Sung et al., 2021). In 2020, there were approximately 19.3 million new cases and 10 million cancer-related deaths worldwide (Sung et al., 2021). Therefore, early diagnosis and effective treatment are critical.

In this study, the expression of CSMD2 was examined and its diagnostic efficacy and prognostic value in pan-cancer were explored. In addition, this study investigated the association between CSMD2 expression and anti-tumor immunity and immune evasion in the tumor microenvironment, and the relationship with immune checkpoints, MHC molecules, and immunostimulators, which clarified the role of CSMD2 in suppressing anti-tumor immunity. Finally, epigenetic methylation analysis and functional enrichment analyses were performed, which provided ideas for further functional experiments.

## 2 Materials and methods

### 2.1 Differential CSMD2 expression analysis in cell lines, normal and tumor tissues

The RNAseq data of TCGA and GTEx was downloaded from UCSC XENA (https://xenabrowser.net/datapages/). The landscape of CSMD2 expression in 33 cancers and corresponding normal tissues were visualized using the ggplot2 package in the R version 4.0.3 program (The R Project for Statistical Computing). Wilcoxon rank sum test was used for analysis.

The Gene Expression Display Server (GEDS) (http://bioinfo.life.hust.edu.cn/web/GEDS/) was used to demonstrate the differential mRNA expression of CSMD2 in normal tissues. Data from Genotype-Tissue Expression (GTEx) was normalized by transcripts per million (TPM) (Xia et al., 2019). The data (log2 (TPM+1)) was from Cancer cell line encylopedia (CCLE) (https://depmap.org/portal/gene/CSMD2?tab=characterization) and visualized by R software for analyzing the expression of CSMD2 in cancer cell lines.

The transcriptome data used for subsequent analyses were exported and downloaded from the GEO database (http://www.ncbi.nih.gov/geo). The raw data were downloaded as MINiML files. Box plots are drawn by boxplot (Zhou et al., 2020). RNA sequencing expression (level 3) profiles and corresponding clinical information for live cancer (Japan) were obtained from the International Cancer Genome Consortium (ICGC) database (https://dcc.icgc.org/releases/current/Projects) (Zhang et al., 2019). Statistical analyses were performed using R software v4.0.3.

The human lung bronchial epithelial cell line BEAS-2B and human non-small cell lung cancer cell line A549 were purchased from the Cell Bank of the Chinese Academy of Sciences (Shanghai, China) and Stem Cell Bank, Chinese Academy of Sciences, respectively. Total RNA from cells was extracted by using TRIzol (Thermo Fisher, Shanghai, China) RNA extraction protocol. Total RNA was reversely transcribed to cDNA using cDNA reverse transcription kits (TransGen Biotech, China). RT-qPCR was performed with TransStart^®^ Top Green qPCR SuperMix (TransGen Biotech, China). GAPDH was used as the internal reference gene for normalization. The 2^−ΔΔCt^ method was used to analyze the qPCR results. The GraphPad Prism (version 8.0) was employed to visualize the relative gene expression levels in cell lines.

### 2.2 Exploring the diagnostic and prognostic potential of CSMD2

The RNAseq data and corresponding clinical data of 33 cancers were downloaded from the TCGA database (https://portal.gdc.cancer.gov/). Receiver operating characteristic (ROC) curves were used to evaluate the diagnostic efficacy of CSMD2. If there were no corresponding paracancerous data in the TCGA database, the tumor tissue data from TCGA and the corresponding normal tissue data from GTEx in UCSC XENA (https://xenabrowser.net/datapages/) would be included. Statistical analysis and visualization were performed via the R software. The pROC package and ggplot2 packages were utilized to calculate the area under the curve (AUC) and visualize the ROC curve.

The closer the AUC is to 1, the better the diagnostic value. AUC between 0.5–0.7 indicates low accuracy, 0.7 to 0.9 indicates moderate accuracy, and greater than 0.9 indicates high accuracy. Univariate Cox regression analysis was performed using the survival package. The Kaplan-Meier Plotter (http://kmplot.com/analysis/) (Győrffy, 2021) was used to show statistically significant results. Oncolnc (http://www.oncolnc.org/) (Anaya, 2016) is a tool for exploring survival correlations, and the cut-off is 50%. The correlation between CSMD2 expression and the clinicopathological stage was visualized via the ggplot2 package.

### 2.3 cBioPortal

“TCGA Pan-Cancer Atlas Studies” in cBioportal (http://www.cbioportal.org) (Cerami et al., 2012; Gao et al., 2013) was employed to explore genetic alteration characteristics of CSMD2. The “Cancer Types Summary” module displayed CSMD2 alteration types and frequency in 32 cancer studies, and the “Mutations” module presented the mutation information of CSMD2. Kaplan-Meier plots with log-rank *p*-values were generated via the “Comparison” module, which can analyze the survival time of cancer patients with or without CSMD2 alterations.

### 2.4 SangerBox

Sangerbox 3.0 website (http://vip.sangerbox.com/home.html) is a visualization tool for bioinformatics analysis. The relationship between CSMD2 expression and tumor mutational burden (TMB), microsatellite instability (MSI), and tumor neoantigen burden (TNB) was analyzed. Their spearman’s correlation was calculated in each type of tumor by the “single gene pan-cancer analysis” module in Sangerbox. CSMD2 expression data were obtained from the TCGA pan-cancer database (PANCAN, N=10535). Simple Nucleotide variation data downloaded from GDC (https://portal.gdc.cancer.gov/) was used to calculate the TMB of samples via R-package “maftools” (version 2.8.05). MSI scores and TNB data for each tumor were obtained from previous studies (Bonneville et al., 2017; Thorsson et al., 2018). Samples with 0 expression levels were filtered, and each expression value was further transformed by log2 (x + 1). Finally, cancer types with fewer than three samples were also eliminated.

### 2.5 UALCAN

UALCAN (http://ualcan.path.uab.edu/analysis.html) (Chandrashekar et al., 2017) was used to analyze the promoter methylation level of CSMD2 between different cancers and the corresponding normal tissues of TCGA samples. The beta value indicated the level of DNA methylation, ranging from 0 (unmethylated) to 1 (fully methylated). Different beta value cut-off values are considered to indicate hypermethylation (beta value: 0.7–0.5) or hypomethylation (beta-value: 0.3–0.25). (Shinawi et al., 2013; Men et al., 2017).

### 2.6 TIMER2.0

TIMER2.0 (http://timer.comp-genomics.org/) (Li et al., 2020) is a comprehensive resource for investigating tumor immunological, clinical, and genomic features of tumors in TCGA. The “Gene_Corr” module was used to investigate the correlation between N6-methyladenosine (m6A) methylation regulators or CSMD2-related genes and CSMD2 expression level. The “Gene” module was used to analyze the relationship between CSMD2 expression and immune infiltration based on two kinds of immune deconvolutions, including CIBERSORT and XCELL. Since most immune cell types are negatively correlated with tumor purity, the “Purity Adjustment”, which used the partial Spearman’s correlation, was selected. The spearman’s rho value indicates the degree of their correlation.

### 2.7 TISIDB

TISIDB (Ru et al., 2019) is an online tool for the network of tumor and immune system interactions. The data were obtained from the PubMed database, high-throughput screening data, exome and RNA sequencing dataset of patient cohorts with immunotherapy, and the TCGA database. The data of correlation between CSMD2 and immunostimulators, and MHC molecules were obtained from TISIDB, and visualized via R (“ggplot” package).

### 2.8 GEPIA2 and STRING

The “Similar Genes Detection” module of GEPIA2 (http://gepia2.cancer-pku.cn/#index) was used to obtain the top 100 CSMD2-related genes and the “Correlation Analysis” module to visualize the correlation between CSMD2 and the top 4 genes in 33 cancers. STRING (version 11.5) (https://www.string-db.org/) was used to predict proteins interacting proteins with CSMD2 and form protein-protein interaction (PPI) network. Functional enrichment analysis of CSMD2 was conducted using the clusterProfiler package, the org. Hs.eg.db package was used for gene ID conversion, and the ggplot2 package used for visualization.

## 3 Results

### 3.1 CSMD2 expression and clinical landscape in pan-cancer

**FIGURE 1:**
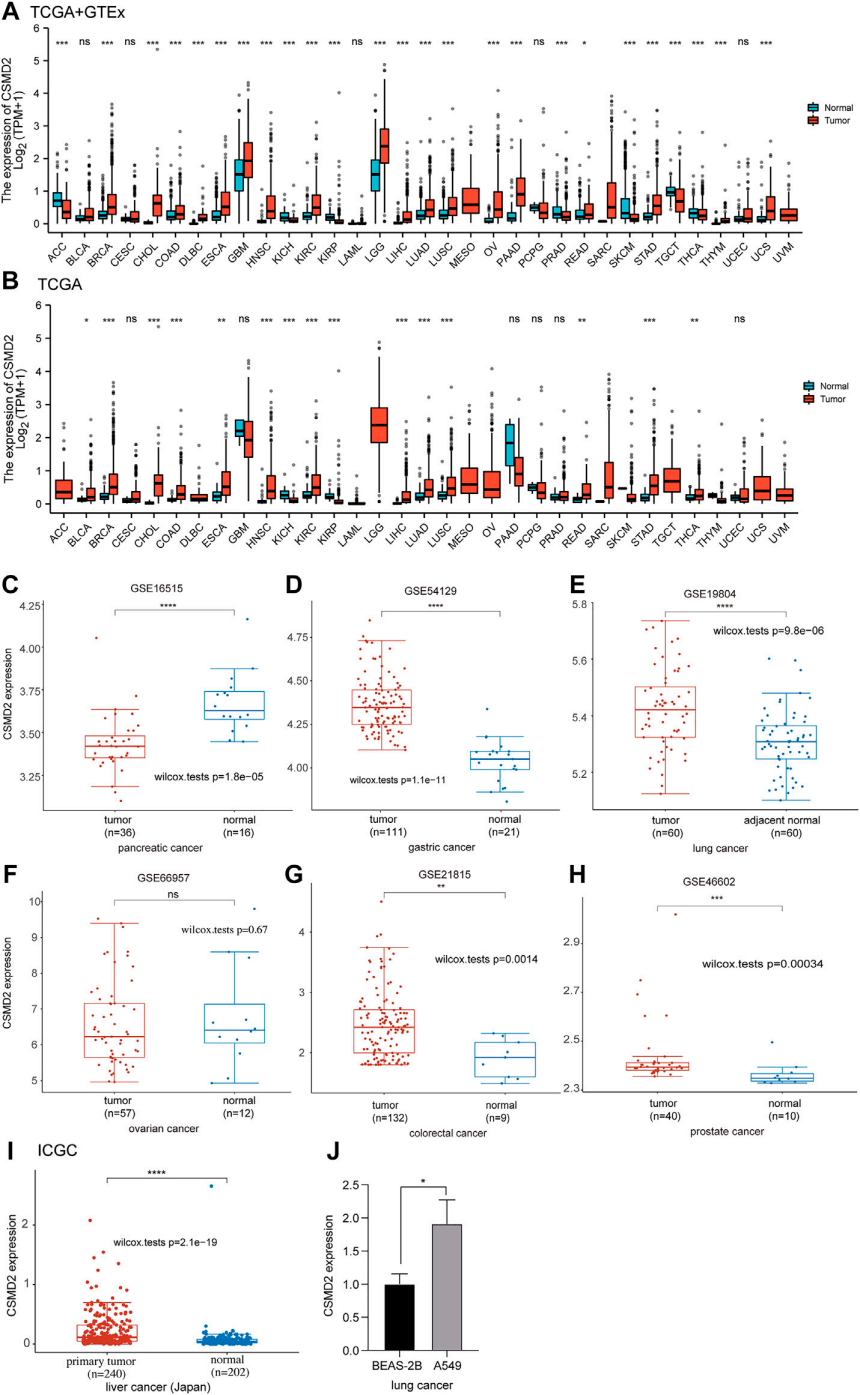
Differential mRNA expression of CSMD2. The boxplot shows CSMD2 expression in **(A,B)** 33 tumor types from TCGA and GTEx databases. The boxplot shows CSMD2 expression in pancreatic cancer **(C)**, gastric cancer **(D)**, lung cancer **(E)**, ovarian cancer **(F)**, colorectal cancer **(G)**, and prostate cancer **(H)** from GEO database. **(I)**The boxplot shows CSMD2 expression in live cancer (Japan) from ICGC databse. **(J)** The expression of CSMD2 in the human lung bronchinal epithelial cell line BEAS-2B and human non-small cell lung cancer cell line A549 (ns, *p* ≥ 0.05; *p < 0.05; **p < 0.01; ***p < 0.001; ****p < 0.0001).

As shown in Figure 1A, CSMD2 was differentially expressed in 25 of the 33 cancers (adrenocortical carcinoma [ACC], breast invasive carcinoma [BRCA], cholangiocarcinoma [CHOL], colon adenocarcinoma [COAD], lymphoid neoplasm diffuse large B-cell lymphoma [DLBC], esophageal carcinoma [ESCA], glioblastoma multiforme [GBM], head-neck squamous cell carcinoma [HNSC], kidney chromophobe [KICH], kidney renal clear cell carcinoma [KIRC], kidney renal papillary cell carcinoma [KIRP], brain lower grade glioma [LGG], liver hepatocellular carcinoma [LIHC], lung adenocarcinoma [LUAD], lung squamous cell carcinoma [LUSC], ovarian serous cystadenocarcinoma [OV], pancreatic adenocarcinoma [PAAD], prostate adenocarcinoma [PRAD], rectum adenocarcinoma [READ], skin cutaneous melanoma [SKCM], stomach adenocarcinoma [STAD], testicular germ cell tumors [TGCT], thyroid carcinoma [THCA], thymoma [THYM], and uterine carcinosarcoma [UCS]). The expression levels of CSMD2 in the tumor tissues of BRCA, CHOL, COAD, DLBC, ESCA, GBM, HNSC, KIRC, LGG, LIHC, LUAD, LUSC, OV, PAAD, READ, STAD, THYM, and UCS were higher than those in normal tissues. Conversely, the expression levels of CSMD2 in the tumor tissues of ACC, KICH, KIRP, PRAD, SKCM, TGCT, and THCA were lower than normal tissues. Figure 1B illustrats the expression of *CSMD2* in 33 tumor types in TCGA database. By analyzing the GEO datasets, we found CSMD2 expressed highly in gastric cancer (*p*-value = 1.1e-11), lung cancer (*p*-value = 9.8e-06), colorectal cancer (*p*-value = 0.0014), and prostate cancer (*p*-value = 0.00034) (Figures 1C–H). As shown in Figure 1I, CSMD2 expressed highly in primary tumor in liver cancer. *In vitro* verification, we also found that CSMD2 expressed highly in human non-small cell lung cancer cell line A549 (Figure 1J). CSMD2 expression in the brain and central nervous system was highest in normal tissues and cancer cell lines (Supplementary Figures S4A,B).

Statistically significant differences in CSMD2 expression were observed based on different pathological stages in BLCA, ESCA, and KIRP, but not in other cancers (Supplementary Figure S1A). Furthermore, CSMD2 expression was significantly correlated with the T or N stage in ESCA, KIRP, and STAD (Supplementary Figure S1B).

**FIGURE 2:**
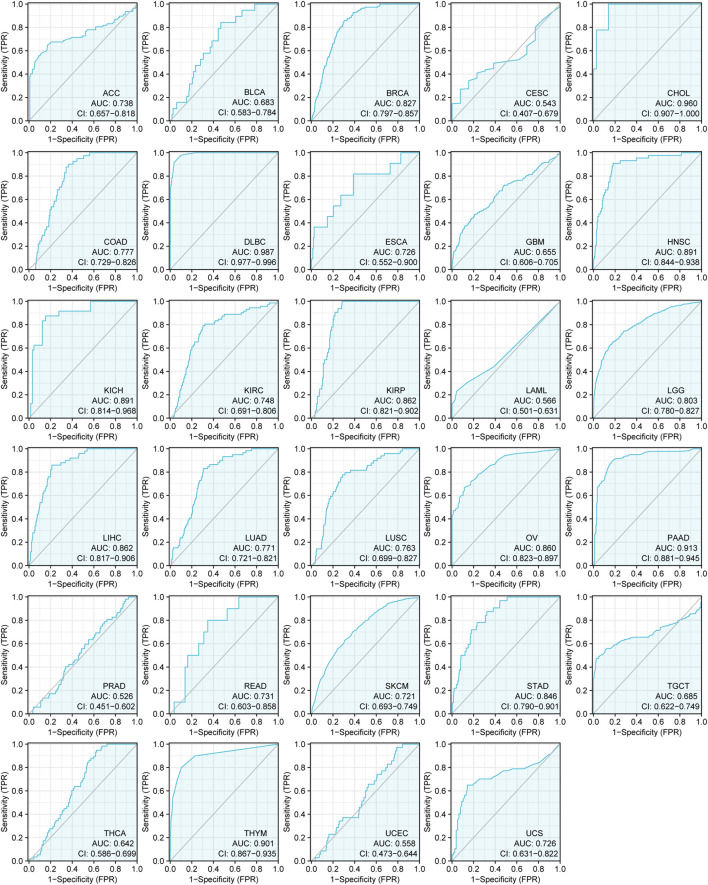
The ROC curves of CSMD2 expression as a diagnostic biomarker in tumor and normal tissues. AUC, area under curve.

We also explored the diagnostic value of CSMD2 as an independent biomarker for pan-cancers. ROC curves were used to evaluate the diagnostic sensitivity and specificity of CSMD2. ROC curves of CSMD2 expression in tumor and normal tissues showed that CSMD2 had high diagnostic efficiency in DLBC (AUC = 0.987, CI: 0.977–0.996), CHOL (AUC = 0.960, CI: 0.907–1.000), PAAD (AUC = 0.913, CI: 0.881–0.945), and THYM (AUC = 0.901, CI: 0.867–0.935), whereas, CSMD2 had low diagnostic efficiency in BLCA, CESC, GBM, LAML, PRAD, THCA, and UCEC, and had moderate diagnostic efficiency in other cancers (Figure 2).

**FIGURE 3:**
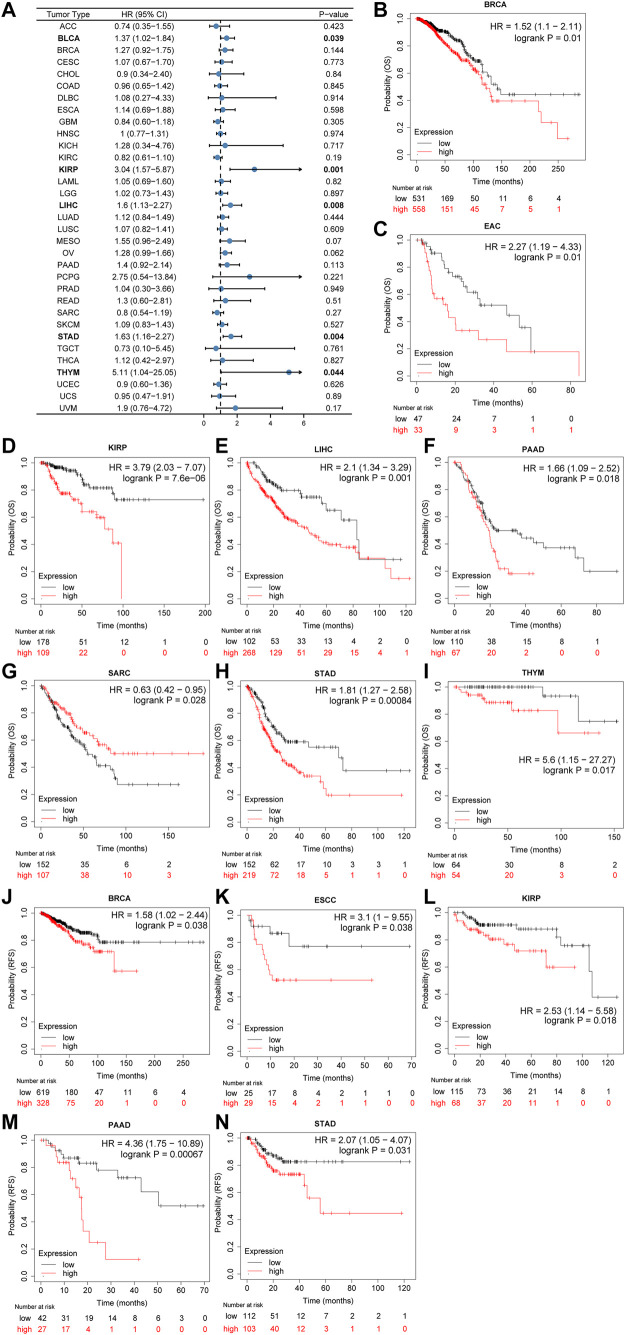
Association of prognosis with CSMD2 expression. **(A)** The forest plots of univariate cox regression analyses for OS. The bold items mean that CSMD2 expression was significantly correlated with prognosis in these types of cancers (*p* < 0.05). **(B–I)** Kaplan–Meier plots for overall survival. CSMD2 overexpression was significantly associated with poor prognosis in BRCA, EAC, KIRP, LIHC, PAAD, SARC, STAD, and THYM patients. **(J–N)** Kaplan–Meier plots for RFS. CSMD2 overexpression was significantly associated with poor prognosis in BRCA, ESCC, KIRP, PAAD, and STAD patients. Items with a hazard ratio greater than 1 indicated that the CSMD2 expression is a promoting factor of death. **(B–N)** were from Kaplan–Meier plotter.

Furthermore, as shown in the forest plots (Figure 3A), a negative association was observed between CSMD2 expression and overall survival (OS) in BLCA, KIRP, LIHC, STAD, and THYM. The results from the Kaplan–Meier plotter demonstrated that CSMD2 overexpression was significantly associated with poor prognosis in patients with BRCA, EAC, KIRP, LIHC, PAAD, SARC, STAD, and THYM patients (Figures 3B–I). Regarding CSMD2 and relapse-free survival (RFS), a significant negative association was found in patients with BRCA, ESCC, KIRP, PAAD, and STAD patients (Figures 3J–N). Patients with high expression of CSMD2 had poor survival in KIRP, LIHC, and STAD (Supplementary Figures S5A–C).

In summary, CSMD2 expression was upregulated in most tumors, with moderate to high diagnostic efficiency, and its high expression was associated with high stage and poor prognosis in tumor patients.

### 3.2 Genetic alteration characteristics of CSMD2

**FIGURE 4:**
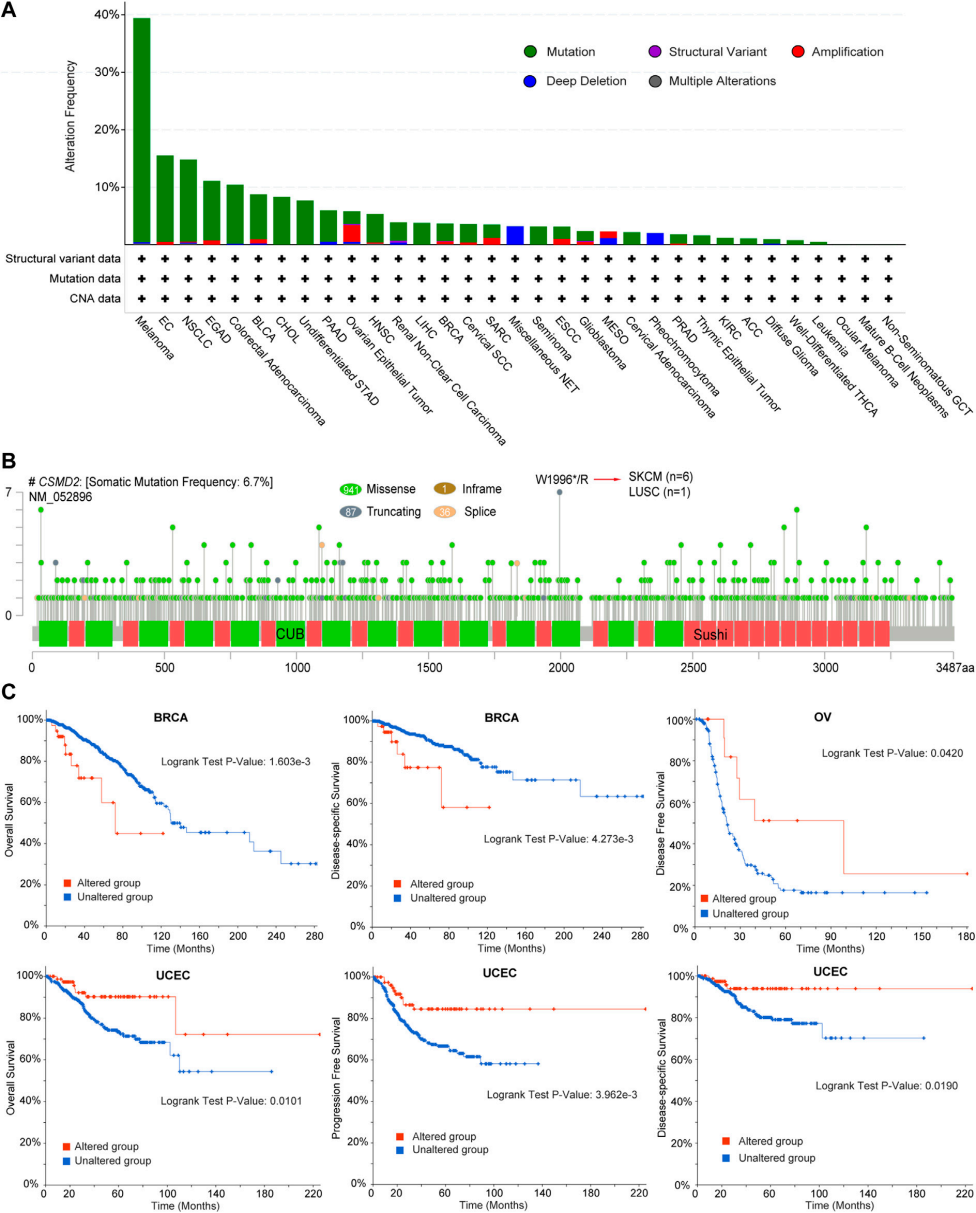
Genetic alteration characteristics of CSMD2. **(A)** Bar plot of CSMD2 alteration frequency and types across different cancer types **(B)** The landscape of CSMD2 mutation with the location, types, and number and their relationship with protein domains, and **(C)** Kaplan-Meier curves of differences in OS, DSS, DFS, and PFS between patients with cancer with and without CSMD2 alteration based on cBioportal. Sushi: Sushi repeat (SCR repeat), CUB: CUB domain.

The frequency and types of genetic alterations of CSMD2 in 32 cancer studies were further investigated. Mutation was the most frequent alteration of CSMD2, whereas structural variation was less frequent (Figure 4A). We further analyzed the number, sites, types, and domains of the CSMD2 mutations. The percentage of samples with somatic mutations in CSMD2 was 6.7%. Missense mutations were the most frequent mutations in CSMD2. The site with the largest number of mutations was W1996*/R in the CUB domain, which was detected in six cases of SKCM and one case of LUSC (Figure 4B). Furthermore, we analyzed the potential relationship between genetic alterations in CSMD2 and the prognosis in patients with different cancer. The results showed that CSMD2 alteration was significantly associated with poor OS (*p*-value = 1.603e-3) and disease-specific survival (DSS) (*p*-value = 4.273e-3) in BRCA patients, favorable disease-free survival (DFS) (*p*-value = 0.0420) in OV patients, and favorable OS (*p*-value = 0.0101), progression-free survival (PFS) (*p*-value = 3.962e-3), and DSS (*p*-value = 0.0190) in UCEC patients (Figure 4C). In summary, the genetic alterations in CSMD2 are related to patient survival.

### 3.3 Correlation of CSMD2 expression with tumor mutational burden, microsatellite instability and tumor neoantigen burden

**FIGURE 5:**
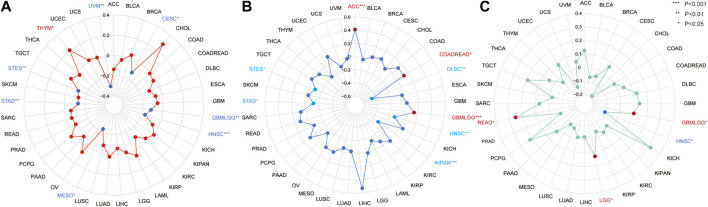
The correlation between the expression of CSMD2 and TMB, MSI, and TNB in tumors. **(A)** The correlation between CSMD2 and TMB is positive in THYM and negative in UVM, CESC, GBMLGG, HNSC, MESO, STAD, and STES **(B)** The correlation between CSMD2 and MSI is positive in ACC, COADREAD, and GBMLGG and negative in DLBC, HNSC, KIPAN, STAD, and STES. **(C)** The correlation between CSMD2 and TNB is negative in HNSC and positive in GBMLGG, LGG, and READ (*, *p* < 0.05; **, *p* < 0.01; ***, *p* < 0.001).

The correlations between CSMD2 expression and TMB, MSI, and TNB were explored and visualized using radar maps. Significant correlations between CSMD2 and TMB were observed in eight tumors (Figure 5A, Supplementary Table S5), including a significant positive correlation in THYM (*p* = 0.033) and negative correlations in GBMLGG (*p* = 0.0025), CESC (*p* = 0.041), STES (*p* = 0.0069), STAD (*p* = 0.006), HNSC (*p* = 0.0002), MESO (*p* = 0.038), and UVM (*p* = 0.0078). Significant correlations between CSMD2 and MSI were observed in eight tumors (Figure 5B, Supplementary Table S4), including positive correlations in GBMLGG (*p* = 0.00013), COADREAD (*p* = 0.023), and ACC (*p* = 0.0002), and negative correlations in STES (*p* = 0.018), KIPAN (*p* = 3.896e-15), STAD (*p* = 0.0188), HNSC (*p* = 0.0065), and DLBC (*p* = 0.001). Significant correlations between CSMD2 and TNB were observed in four tumors (Figure 5C, Supplementary Table S3), including positive correlations in GBMLGG (*p* = 0.0398), LGG (*p* = 0.023), and READ (*p* = 0.033), and a negative correlation in HNSC (*p* = 0.043).

### 3.4 Epigenetic methylation analysis

**FIGURE 6:**
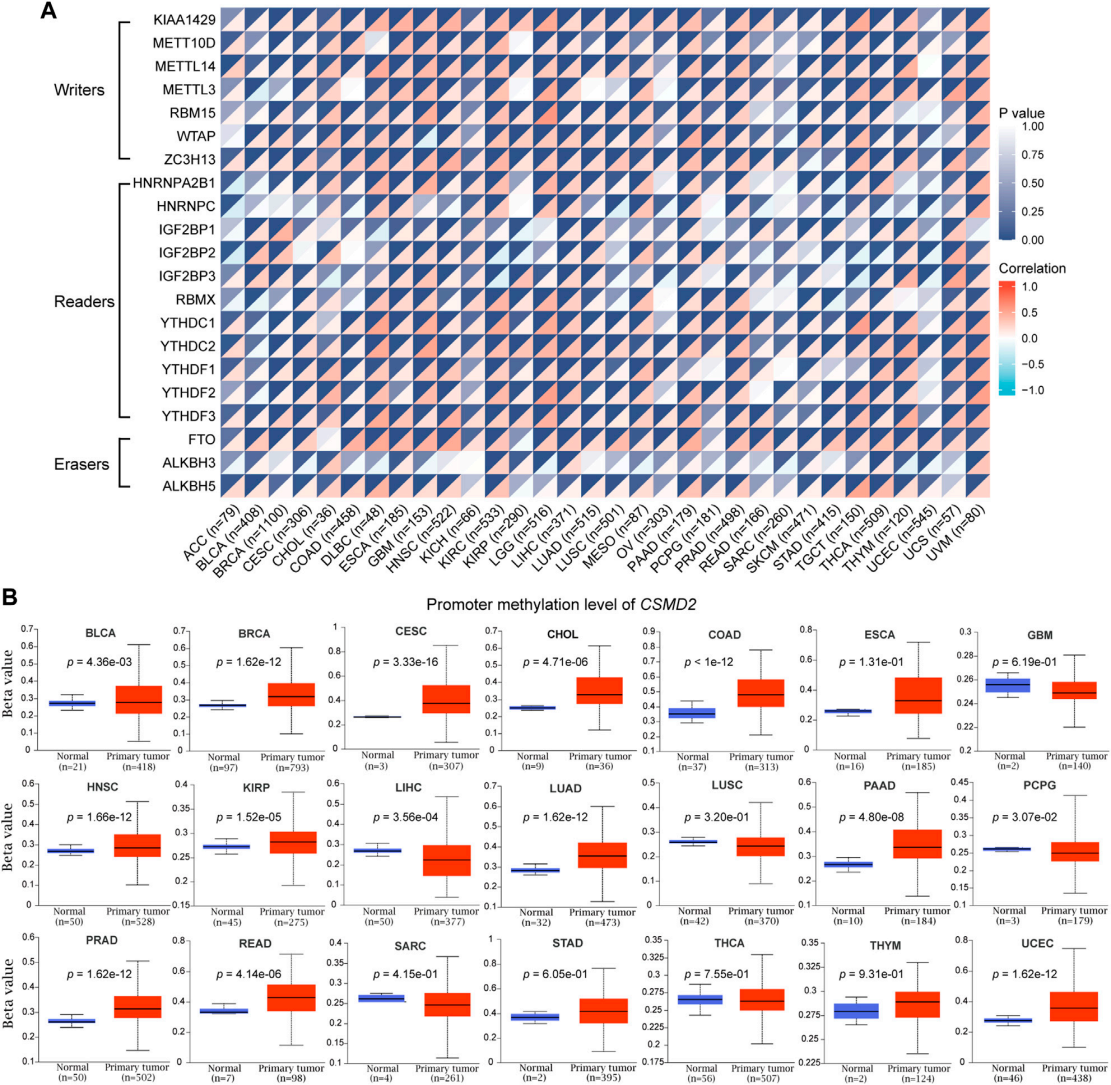
Epigenetic methylation analysis of CSMD2. **(A)** The correlation between the expression of CSMD2 mRNA and m6A methylation regulatory factors in multiple cancers. Correlations are depicted with Spearman’s rho values and statistical significance. **(B)** Differential promoter methylation level (beta values) of CSMD2 in normal tissues and tumors based on UALCAN.

Considering that m6A methylation plays an important role in tumorigenesis and development, the correlation between the expression of CSMD2 mRNA and m6A methylation regulatory factors was investigated for multiple cancers. A total of 21 key m6A methylation regulators, including seven writers (KIAA1429, METT10D, METTL14, METTL3, RBM15, WTAP, and ZC3H13), 11 readers (HNRNPA2B1, HNRNPC, IGF2BP1, IGF2BP2, IGF2BP3, RBMX, YTHDC1, YTHDC2, YTHDF1, YTHDF2, and YTHDF3) and three erasers (FTO, ALKBH3, ALKBH5) were selected. The heatmap indicated that CSMD2 mRNA was positively correlated with most m6A methylation regulatory factors in most cancers (Figure 6A). Additionally, promoter methylation levels of CSMD2 in normal tissues and tumors were compared. CSMD2 is hypermethylated in various cancers, including BLCA, BRCA, CESC, CHOL, COAD, HNSC, KIRP, LUAD, PAAD, PRAD, READ, and UCEC. In contrast, it is hypomethylated in LIHC and PCPG (Figure 6B).

In summary, hypermethylation of CSMD2 promoter and high levels of m6A methylation regulators have been observed in most cancers.

### 3.5 Immune infiltration, immune evasion, and immune checkpoints analysis in the tumor microenvironment

**FIGURE 7:**
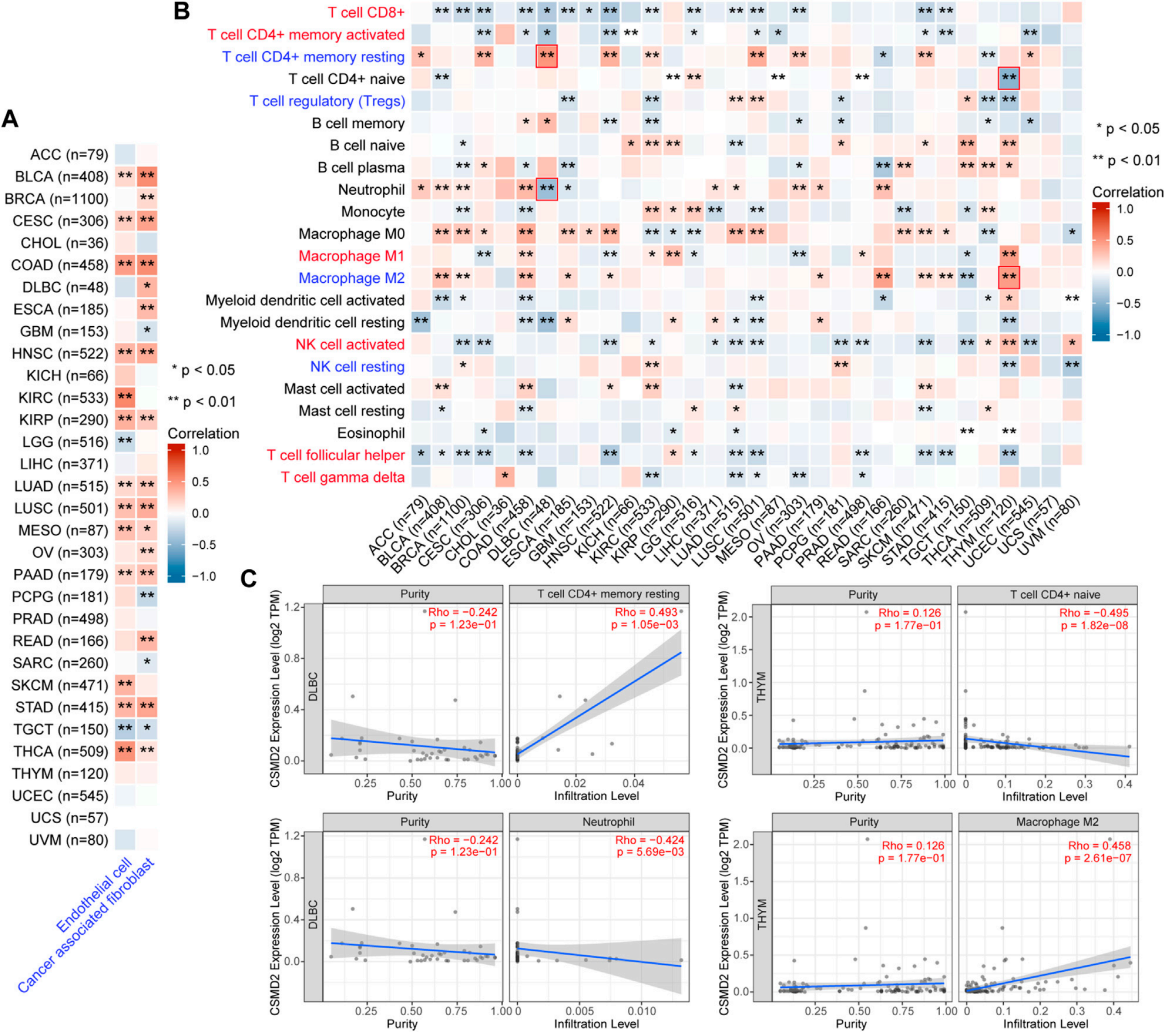
Correlation analysis between CSMD2 expression and immune infiltration in the tumor microenvironment. Heat maps display a correlation between CSMD2 expression level and infiltration level of **(A)** cancer-associated fibroblasts and endothelial cells based on XCELL, and **(B)** 22 immune cells based on CIBERSORT in pan-cancer. Anti-tumor immune cells are highlighted in red, and immune evasion-associated cells are highlighted in blue. **(C)** The scatter plots show the top two strongest positive and negative correlations marked in red boxes in **(B)**. Correlations are depicted with the partial Spearman’s correlation and statistical significance based on TIMER2.0.

The correlation between CSMD2 expression and immune cells in the tumor microenvironment was investigated. The levels of cancer-associated fibroblasts (CAFs) and endothelial cells were positively correlated with CSMD2 expression in most cancers (Figure 7A). CSMD2 expression was positively correlated with the infiltration of anti-tumor immune cells (CD8^+^ T cells, activated memory CD4^+^ T cells, M1 macrophages, activated NK cells, follicular helper T cells, and gamma delta T cells) in CHOL, KIRP, THYM, and UVM, and negatively correlated with immune evasion associated cells (resting memory CD4^+^ T cells, T cell regulatory (Tregs), M2 macrophages and resting NK cells). In contrast, it was negatively correlated with the infiltration of anti-tumor immune cells and positively correlated with immune evasion associated cells in most other cancers (Figure 7B). The top two strongest positive correlations with CSMD2 expression were the infiltration level of resting memory CD4^+^ T cells in DLBC (*p* = 1.05e-03) and M2 macrophages in THYM (*p* = 2.61e-07). The top two strongest negative correlations with CSMD2 expression are the infiltration level of naive CD4^+^ T cells in THYM (*p* = 1.82e-08) and neutrophils in DLBC (*p* = 5.69e-03) (Figure 7C).

**FIGURE 8:**
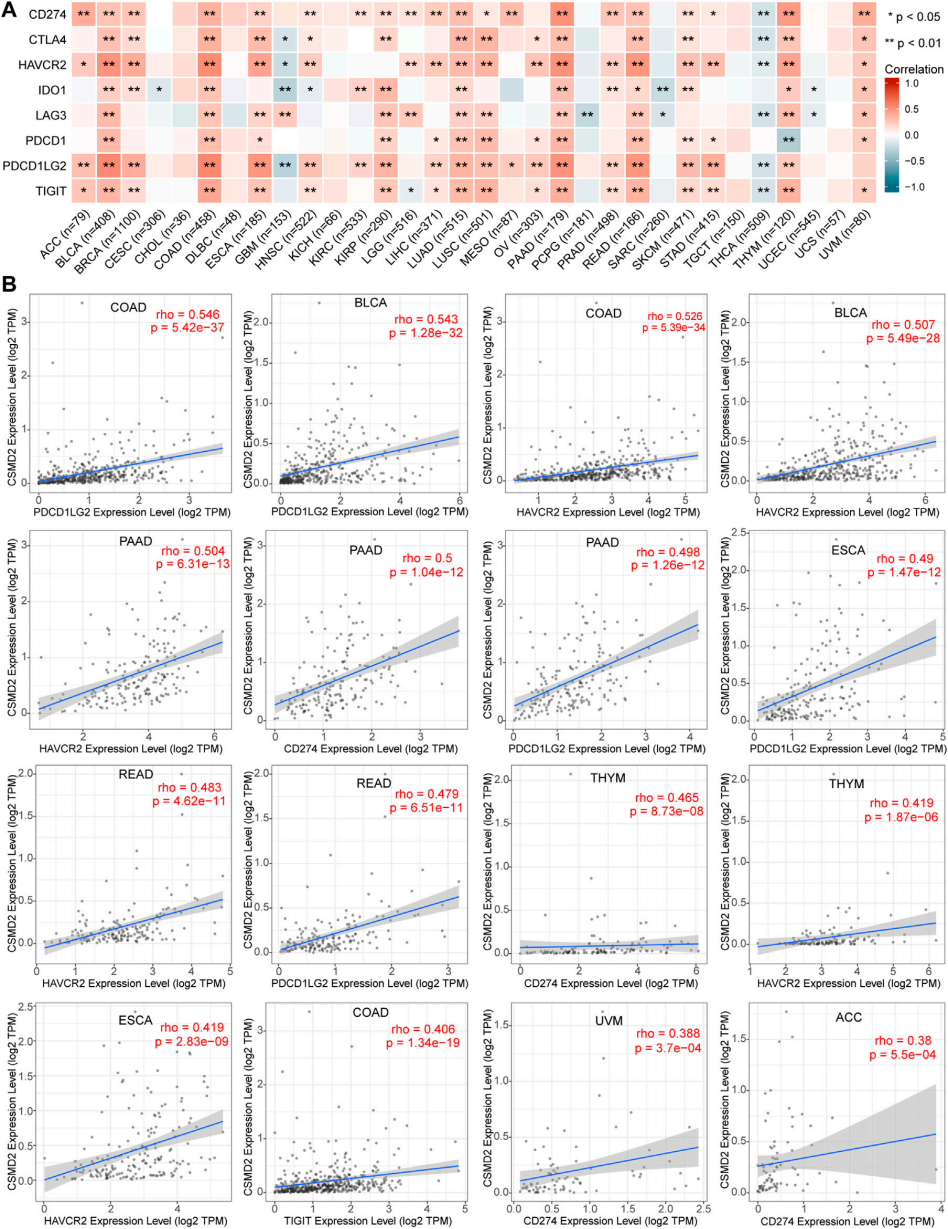
Correlation analyses of the CSMD2 expression with immune checkpoints. **(A)** Heat maps display a correlation between CSMD2 expression and immune checkpoints, including TIGIT, CD274, PDCD1, LAG3, HAVCR2, CTLA4, IDO1, and PDCD1LG2 in pan-cancer. **(B)** The top sixteen strongest correlations are displayed via scatter plots. Correlations are depicted with Spearman’s correlation and statistical significance based on TIMER 2.0.

Since tumor cells take advantage of immune checkpoints to evade immune responses, the relationships between the CSMD2 expression and the most common immune checkpoints, including TIGIT, CD274, PDCD1, LAG3, HAVCR2, CTLA4, IDO1, and PDCD1LG2 was also analyzed. The expression of CSMD2 was positively correlated with most immune checkpoints in ACC, BLCA, BRCA, COAD, ESCA, HNSC, KIRC, KIRP, LGG, LIHC, LUAD, LUSC, OV, PAAD, PRAD, READ, SKCM, STAD, THYM, and UVM. In contrast, the expression of CSMD2 negatively correlated with most immune checkpoints in GBM, PCPG, SARC, THCA, and UCEC. Notably, PDCD1LG2, HAVCR2, and CD274 showed the strongest positive correlations with CSMD2 expression in most tumors. (Figures 8A,B).

We observed that the expression of CSMD2 was negatively correlated with MHC-I molecules in CESC, GBM, LGG, PCPG, SARC, STAD, TGCT, and THCA but positively correlated with MHC-I molecule in BLCA, COAD, and LUSC (Supplementary Figure S2). The correlation between the expression of CSMD2 and MHC-II was positive in BLCA, BRCA, COAD, LUSC, OV, PAAD, READ, and SKCM but negative in GBM and THCA (Supplementary Figure S2).

Immunostimulators mainly work at various stages of lymphoid differentiation, development, and maturation, and regulate immune function, thereby enhancing the ability of the body to prevent and resist disease and exert an anti-tumor role. We observed that CSMD2 was positively correlated with immunostimulators in most tumors, whereas it was negatively correlated with immunostimulators in GBM and THCA (Supplementary Figure S3).

In most cancers, CSMD2 was associated with immune evasion or immunosuppression. Additionally, there was insufficient anti-tumor immunity and up-regulation of immune checkpoints.

### 3.6 Functional enrichment analysis

**FIGURE 9:**
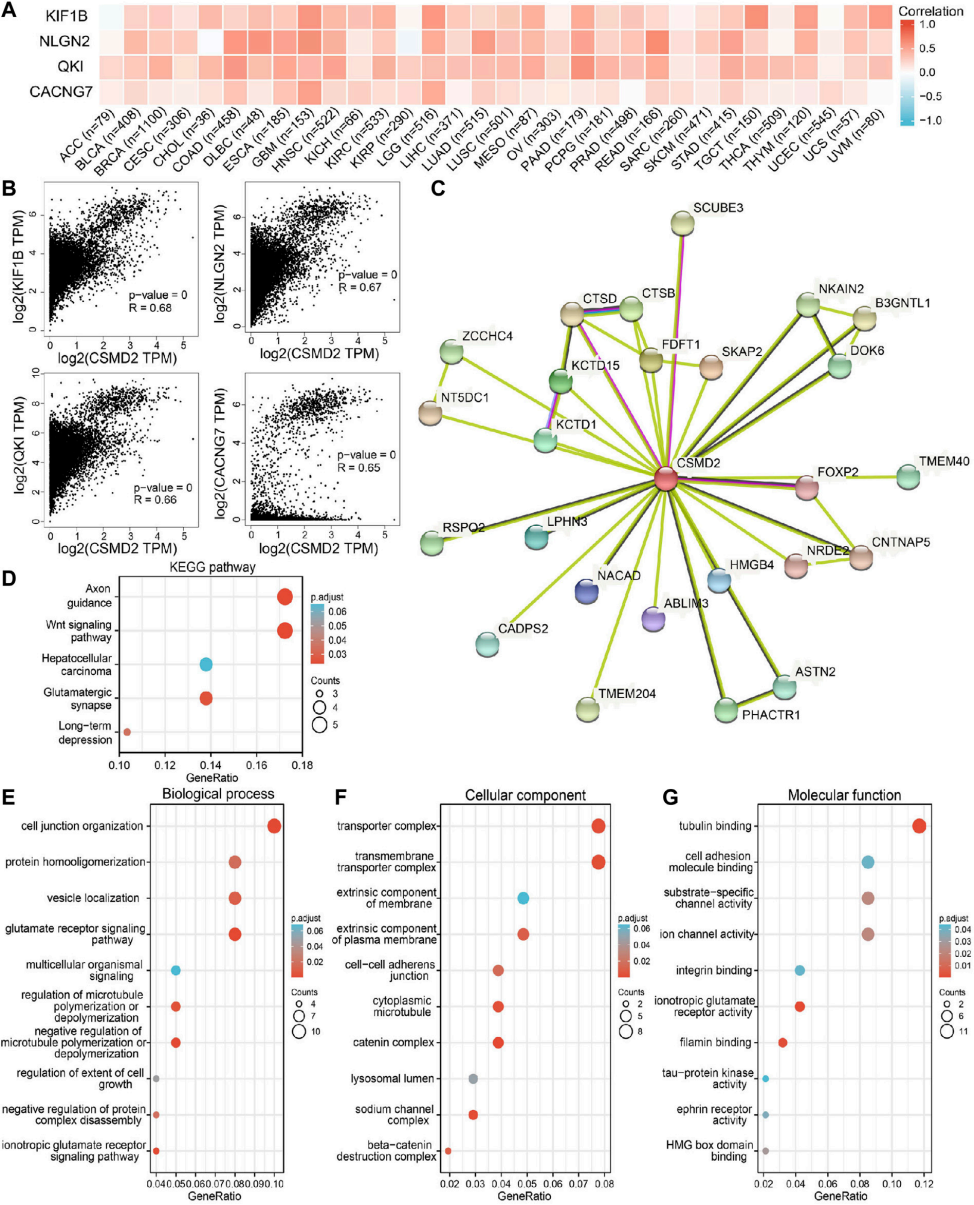
CSMD2-related genes, interacting proteins and functional enrichment analysis. **(A)** and **(B)** showed the correlation between CSMD2 and the top four genes related to it in 33 cancers. **(C)** The PPI network of CSMD2. GO analyses, including **(D)** KEGG pathway, **(E)** biological process, **(F)** cellular component, and **(G)** molecular function.

To further explore the biological function of CSMD2 in pan-cancer, a series of enrichment analyses were performed. The top 100 genes (Supplementary Table S1) associated with CSMD2 were obtained, and the top four genes including *KIF1B*, *NLGN2*, *QKI* and *CACNG7,* are displayed in Figures 9A,B. Additionally, 25 predicted proteins interacted directly with CSMD2 displayed in the PPI network, of which the interactions with CTSD, SCUBE3 and FOXP2 were experimentally determined (Figure 9C). Both the top 100 CSMD2-related genes and 25 CSMD2-interacting proteins were included in the functional enrichment analysis. We finally obtained 238 gene ontology (GO) terms including 133 biological processes (BP) terms, 75 cellular components (CC) terms, 34 molecular functions (MF) terms and 5 KEGG pathways (Supplementary Table S2). The KEGG analysis results confirmed the enrichment of Wnt signaling pathway and hepatocellular carcinoma pathway (Figure 9D). Furthermore, GO terms related to cancers were displayed. BP analysis showed that CSMD2 might be associated with the glutamate receptor signaling pathway, negative regulation of microtubule polymerization or depolymerization, cell junction organization, protein homooligomerization, and negative regulation of protein complex disassembly, regulation of extent of cell growth, and multicellular organismal signaling (Figure 9E). CC analysis revealed that CSMD2-related genes were enriched in the transmembrane transporter complex, transporter complex, cytoplasmic microtubule, extrinsic component of the plasma membrane, cell-cell adherens junction, and lysosomal lumen (Figure 9F). MF analysis showed that the related genes were associated with tubulin binding, ion channel activity, substrate-specific channel activity, HMG box domain binding, cell adhesion molecule binding, integrin binding, and tau-protein kinase activity (Figure 9G).

## 4 Discussion

Few studies have investigated the effects of CSMD2 on tumorigenesis and development and its molecular mechanisms. To the best of our knowledge, this is the first study to explore the role of CSMD2 in pan-cancers. The results showed that the expression of CSMD2 was inconsistent in 33 types of cancers, of which CSMD2 expression was upregulated in most tumors. Meanwhile, we analyzed CSMD2 expression in tumors from GEO and ICGC database. It had moderate or high diagnostic efficacy, and the high expression was related to a higher stage and poor prognosis, for example, the expression level of CSMD2 was high in gastric cancer, and patients with high CSMD2 expression had poor prognosis. These results suggested that CSMD2 may be an oncogenic molecule involved in tumorigenesis and development.

Missense mutations were the most common type of CSMD2 alterations. Seven mutations were detected in the W1996*/R site of the CUB domain, which were detected in six cases of SKCM and one case of LUSC. Whether this is a functional mutation site remains to be further verified. Further clinical correlation analysis showed that CSMD2 alterations were related to the survival of patients with cancer. CSMD2 alterations were associated with poor survival in BRCA and better survival in patients with OV and UCEC.

There is growing evidence that epigenetic modifications play a vital role in tumors through various mechanisms, in which m6A methylation is a common type of RNA modification. RNA methylation is regulated by different types of regulatory factors, including methyltransferases (writers), RNA-binding proteins (readers) and demethylases (erasers) (Yang Y. et al., 2018; Li et al., 2019). The level of m6A methylation was indirectly known by investigating the levels of these regulatory factors. Our results showed that CSMD2 expression was positively correlated with m6A methylation regulatory factors, and implied that m6A methylation levels might be positively correlated with CSMD2 expression in pan-cancer.

TMB and TNB are biomarkers for therapeutic benefits in many tumors (Hollern et al., 2019). The anti-tumor immune response is likely related to high TMB(Pakish et al., 2017). Usually, the higher the mutation burden, the higher the possibility of neoantigens, and hence, the higher the immunotherapy response rate (Turajlic et al., 2017). A defective DNA mismatch repair (MMR) system leads to the accumulation of genetic errors while copying microsatellite loci, resulting in MSI(de Rosa et al., 2016). High microsatellite instability (MSI-H) had been used as a biomarker of the impaired function of the MMR system and is correlated with better efficacy of immunotherapy (Lee et al., 2002; Chen et al., 2019). Higher TMB is reportedly associated with better OS and better response to ICIs(Samstein et al., 2019). In this study, CSMD2 expression was found to be negatively correlated with TMB and MSI in both STAD and STES. CSMD2 expression negatively correlated with TMB, MSI, and TNB in HNSC. CSMD2 was highly expressed in the above three tumors, and high CSMD2 expression was correlated with poor prognosis in patients with STAD. Through the correlation analysis of CSMD2 with TMB, MSI, and TNB, we predicted that CSMD2 might play a role in immunotherapy.

MHC-I molecules present endogenous antigens and activate CD8^+^ T-cells, which are then transformed into active cytotoxic T lymphocytes to kill target cells. MHC-II molecules are mainly involved in presenting exogenous antigenic peptides to CD4^+^ T cells, which activate CD4^+^ helper T cells, proliferates and express the corresponding lymphokines, and initiate humoral immune responses. In this study, CSMD2 was negatively correlated with MHC-I molecules, whereas, it was positively correlated with MHC-II molecules, and immunostimulators in most tumors. The mechanism of tumor immune regulation is highly complex, therefore, the relationship between CSMD2 and immunity requires further research.

Tumor microenvironment is the surrounding environment where tumor develops and survives. In addition to tumor cells, surrounding fibroblasts, immune and inflammatory cells, and microvessels are present in the TME (Hui and Chen, 2015). We observed that CSMD2 expression was negatively correlated with infiltration of anti-tumor immune cells, including CD8^+^ T cells, activated memory CD4^+^ T cells, M1 macrophages, activated NK cells, follicular helper T cells, and gamma delta T cells, as well as positively correlated with immune evasion- or suppression-associated cells, including CAFs, endothelial cells, Tregs, and M2 macrophages in most cancers. Therefore, CSMD2 is speculated to promote tumor cells proliferation, migration and invasion through immune escape or immunosuppression rather than anti-tumor immune infiltration. In addition, the upregulation of immune checkpoints helps explain this and may provide the possibility of promoting ICI effects in patients with cancer.

In this study, the correlation between CSMD2 expression and anti-tumor immune response and microenvironment was comprehensively analyzed. It was found that CSMD2 expression might be related to immune escape and promoting the occurrence and development of tumors. The major challenge of current cancer immunotherapy is specific tumor immune response. However, tumors of different types and sites vary in their response to immunotherapy, the mechanism is extremely complex, which needs to be studied in future.

Evaluating global methylation abnormalities by methylation load can predict the degree of tumor immunogenicity. The degree of abnormal methylation is negatively correlated with tumor immunogenicity (Park et al., 2021). High levels of promoter methylation of CSMD2 and m6A methylation were found in tumor tissues or high CSMD2 expression tissues, suggesting the importance of abnormal methylation in tumor evasion of immune surveillance.

Enrichment analyses showed that CSMD2 might be located on the cell membrane, constitute a component of channel proteins, and participate in signal transduction between tumor cells. Interestingly, pathway enrichment analyses revealed its relation to the Wnt signaling pathway and hepatocellular carcinoma pathway. Therefore, further experimental verification is required to confirm this finding. In addition, the predicted proteins interacting with CSMD2 need to be verified.

## Data Availability

The original contributions presented in the study are included in the article/Supplementary Material, further inquiries can be directed to the corresponding author.
