# Supplementary material for: Integrated pan-cancer analysis of CSMD2 as a potential prognostic, diagnostic, and immune biomarker
Source: Front Genet. 2022 Aug 17;13:918486. doi: 10.3389/fgene.2022.918486 (PMC9428318; doi:10.3389/fgene.2022.918486)
Supplement: Supplementary file 1 [file DataSheet1.zip › Supplementary materials-2022Aug5/Supplementary_Material.docx]

Supplementary Material

# Supplementary Figures and Tables

## Supplementary Figures


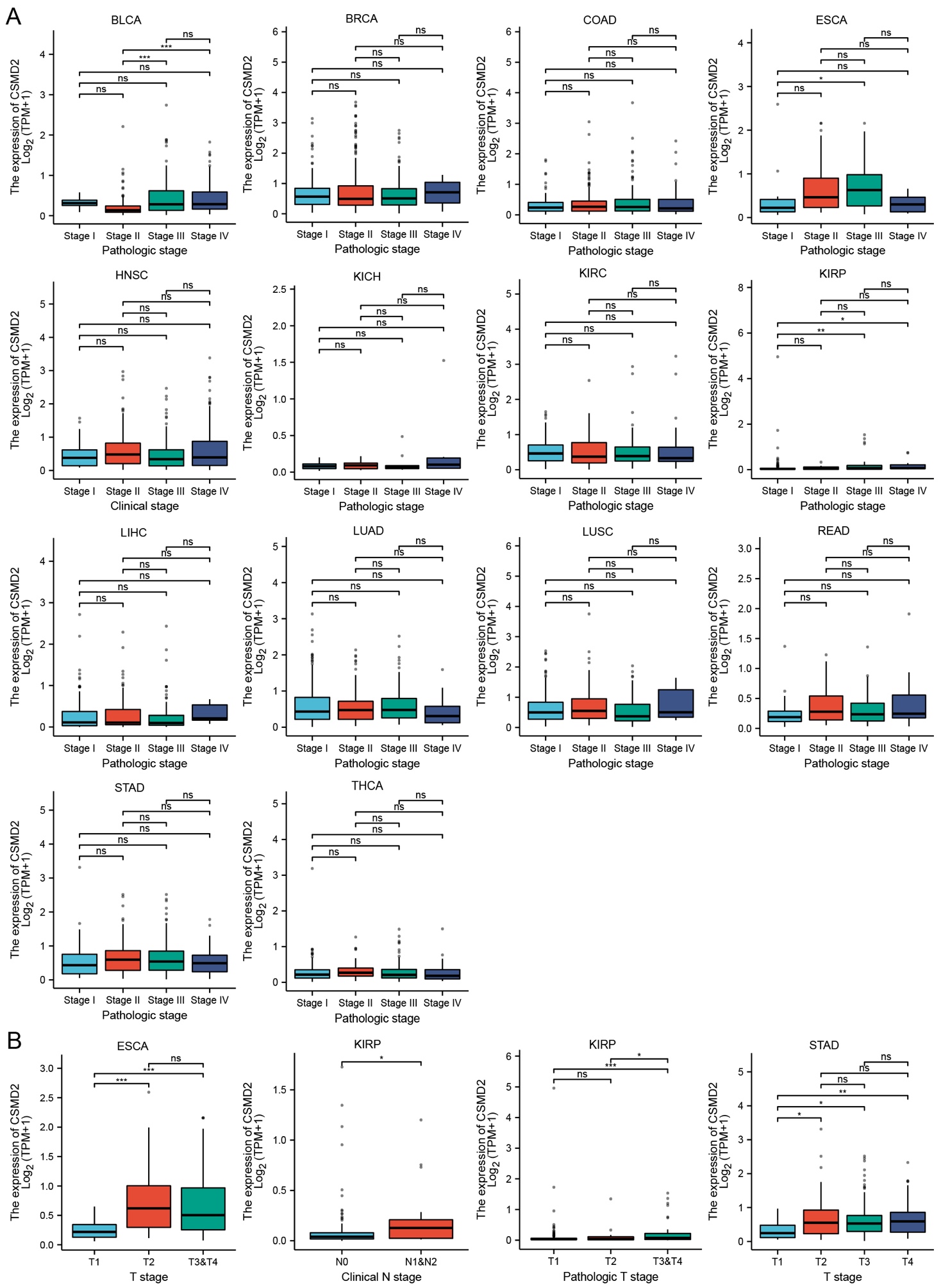


**Supplementary Figure S1.** The association of clinical or pathological stage with CSMD2 expression. (A) CSMD2 expression was significantly correlated with pathological stage in BLCA, ESCA and KIRP, and (B) with T or N stage in ESCA, KIRP, and STAD (ns, p ≥ 0.05; *, p < 0.05; **, p < 0.01; ***, p < 0.001).

**Supplementary Figure S2.** The correlation between CSMD2 and MHC molecule in tumors (*, p < 0.05; **, p < 0.01).

**Supplementary Figure S3.** The correlation between CSMD2 and immunostimulator in tumors (*, p < 0.05; **, p < 0.01).


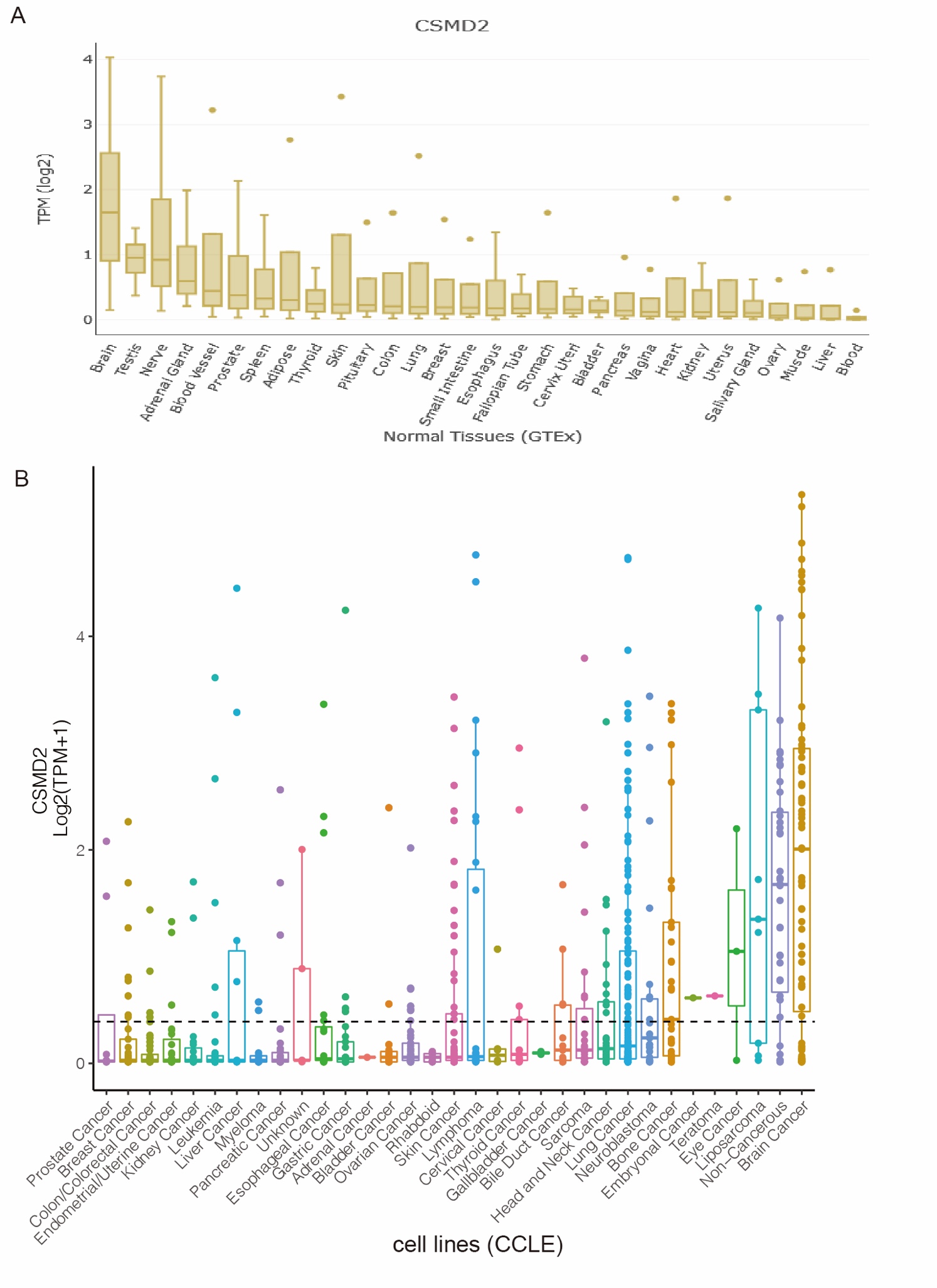


**Supplementary Figure S4.** The boxplot shows CSMD2 expression in normal tissues (A) from GTEx and cancer cell lines (B) from CCLE.

**
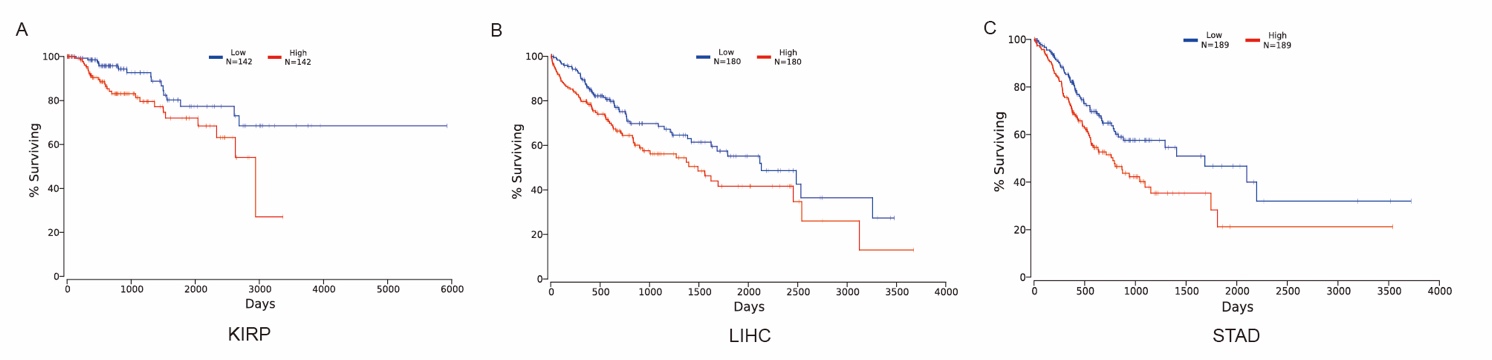
**

**Supplementary Figure S5.** The association of prognosis with CSMD2 expression from Oncolnc. Patients with high CSMD2 expression had poor survival in (A) KIRP，(B)LIHC, and (C) STAD.

## Supplementary Tables

**Supplementary Table S1.** The top 100 genes associated with *CSMD2*.

**Supplementary Table S2.** GO terms and KEGG pathways enriched.

**Supplementary Table S3.** The correlation between the expression of CSMD2 and TNB.

**Supplementary Table S4.** The correlation between the expression of CSMD2 and MSI.

**Supplementary Table S5.** The correlation between the expression of CSMD2 and TMB.

**Supplementary Table S6.** The correlation between CSMD2 and MHC molecules

**Supplementary Table S7.** The correlation between the expression of CSMD2 and immunostimulators.

**Supplementary Table S8.** The expression level of CSMD2 in tumors and their normal tissues from TCGA and GTEx databases.

**Supplementary Table S9.** The expression level of CSMD2 in tumors from TCGA database.

**Supplementary Table S10.** The primer sequences of CSMD2

|  | primer sequences |
| --- | --- |
| CSMD2-F | TACATCGGTCCCGGATCTCA |
| CSMD2-R | CTGCCCTGCTCGATCTCTTC |
