# Supplementary figures and images for: Integrated pan-cancer analysis of CSMD2 as a potential prognostic, diagnostic, and immune biomarker
Source: Front Genet. 2022 Aug 17;13:918486. doi: 10.3389/fgene.2022.918486 (PMC9428318; doi:10.3389/fgene.2022.918486)

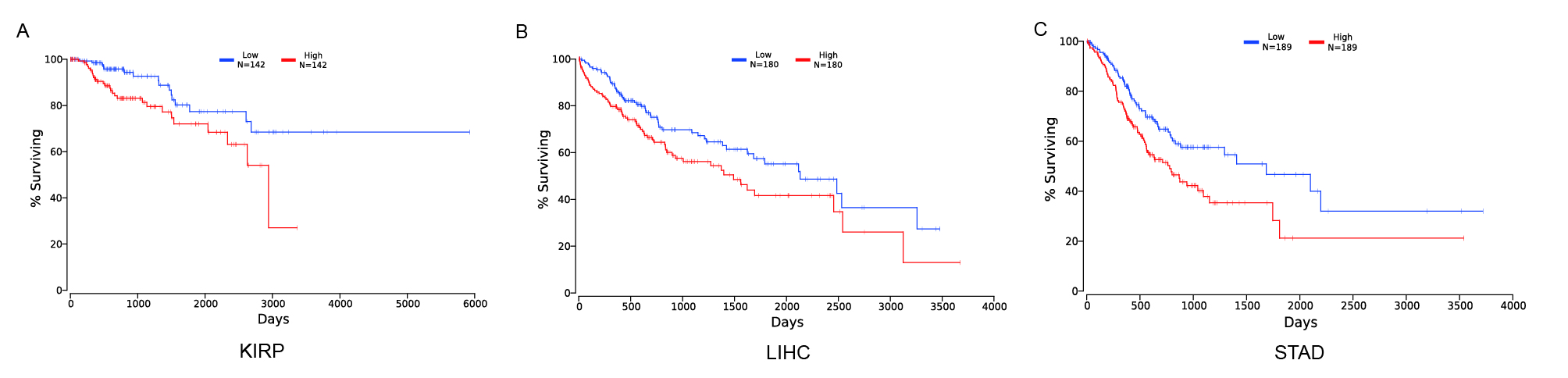

Supplement: Supplementary file 1 [file DataSheet1.zip › Supplementary materials-2022Aug5/Supplementary Figure S5. The association of prognosis with CSMD2 expression from Oncolnc..jpg]

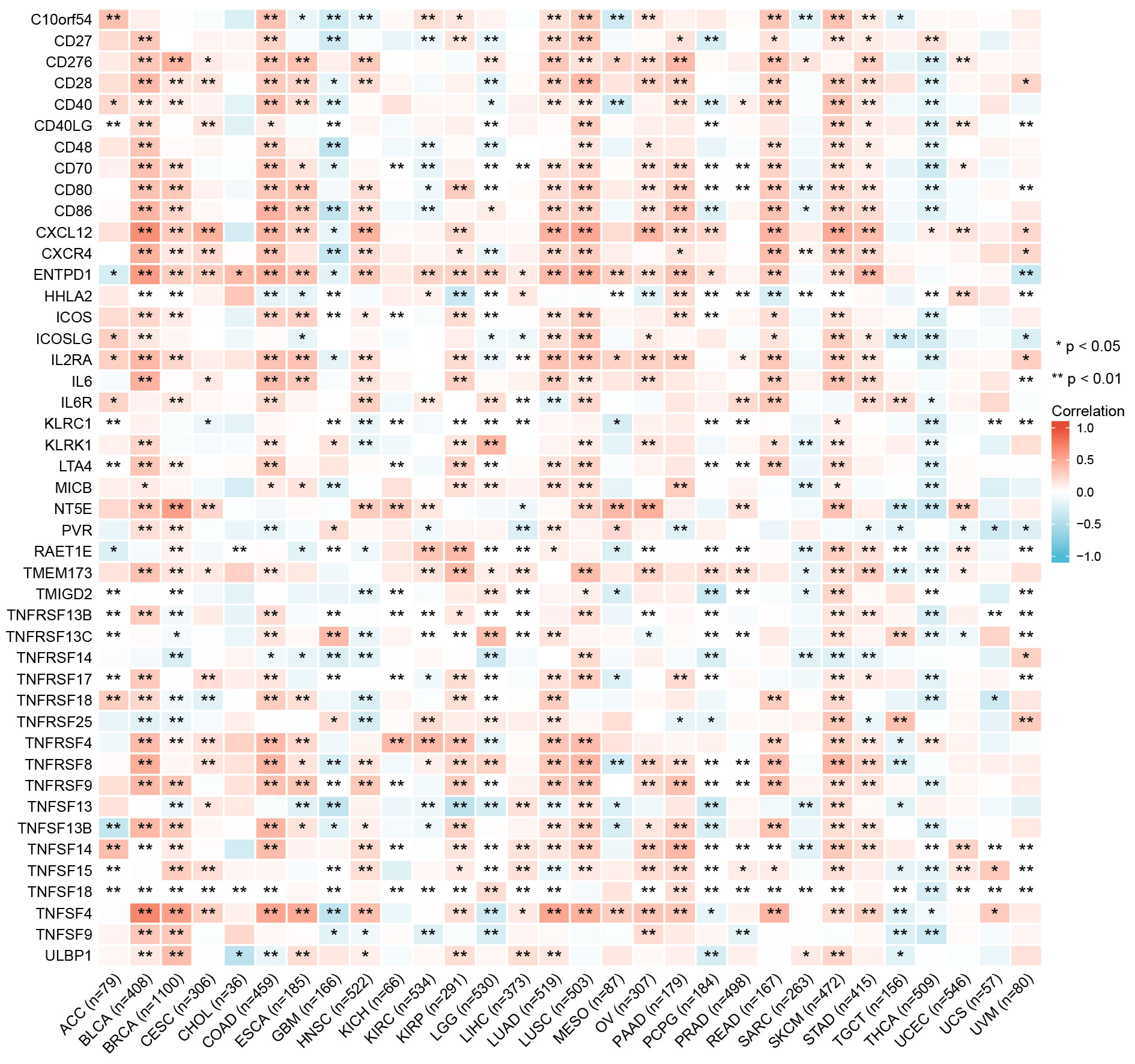

Supplement: Supplementary file 1 [file DataSheet1.zip › Supplementary materials-2022Aug5/Supplementary Figure S3. The correlation between CSMD2 and immunostimulator in tumors.jpg]

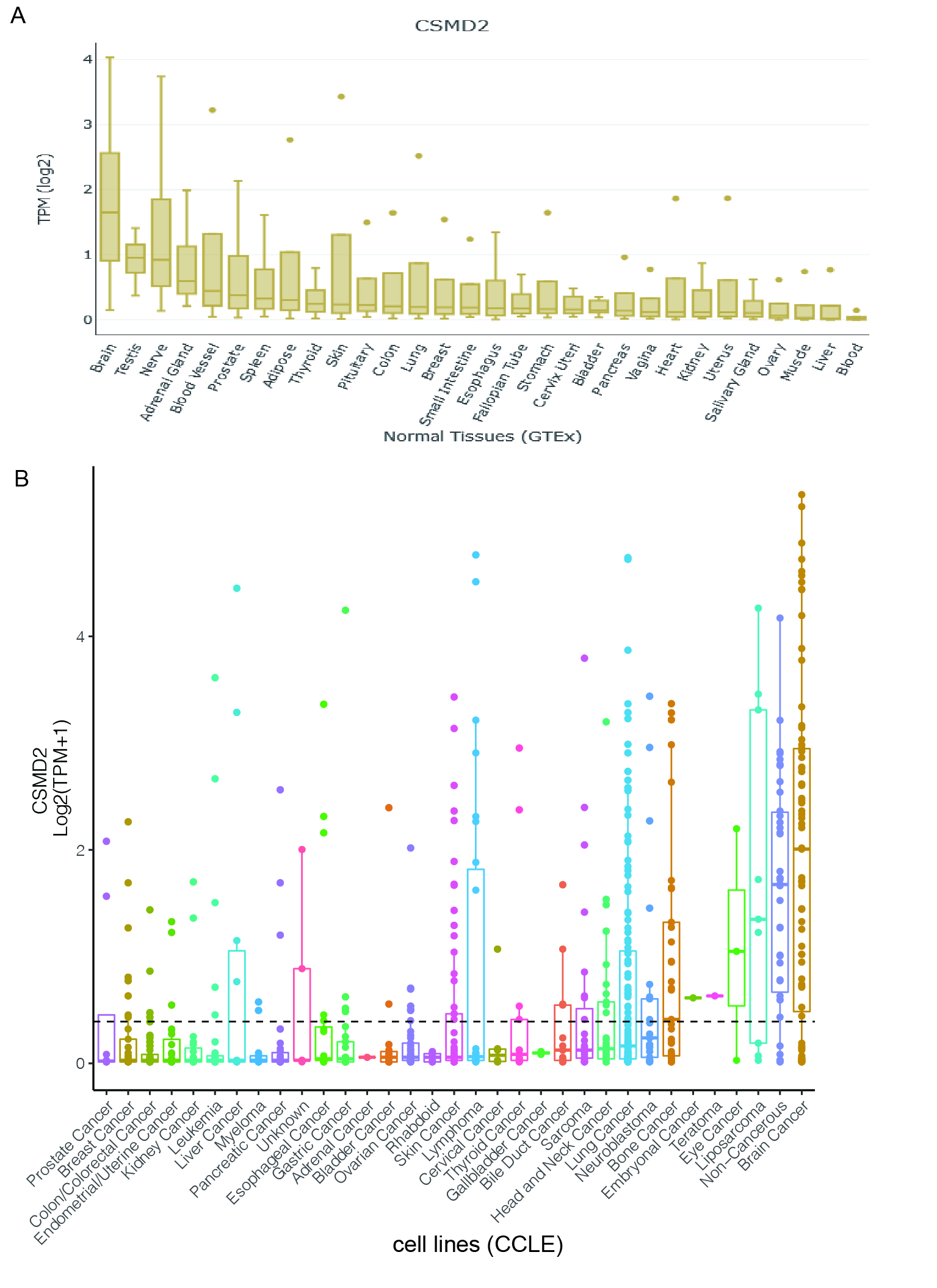

Supplement: Supplementary file 1 [file DataSheet1.zip › Supplementary materials-2022Aug5/Supplementary Figure S4. The boxplot shows CSMD2 expression in normal tissues (A) from GTEx and cancer cell lines (B) from CCLE.jpg]

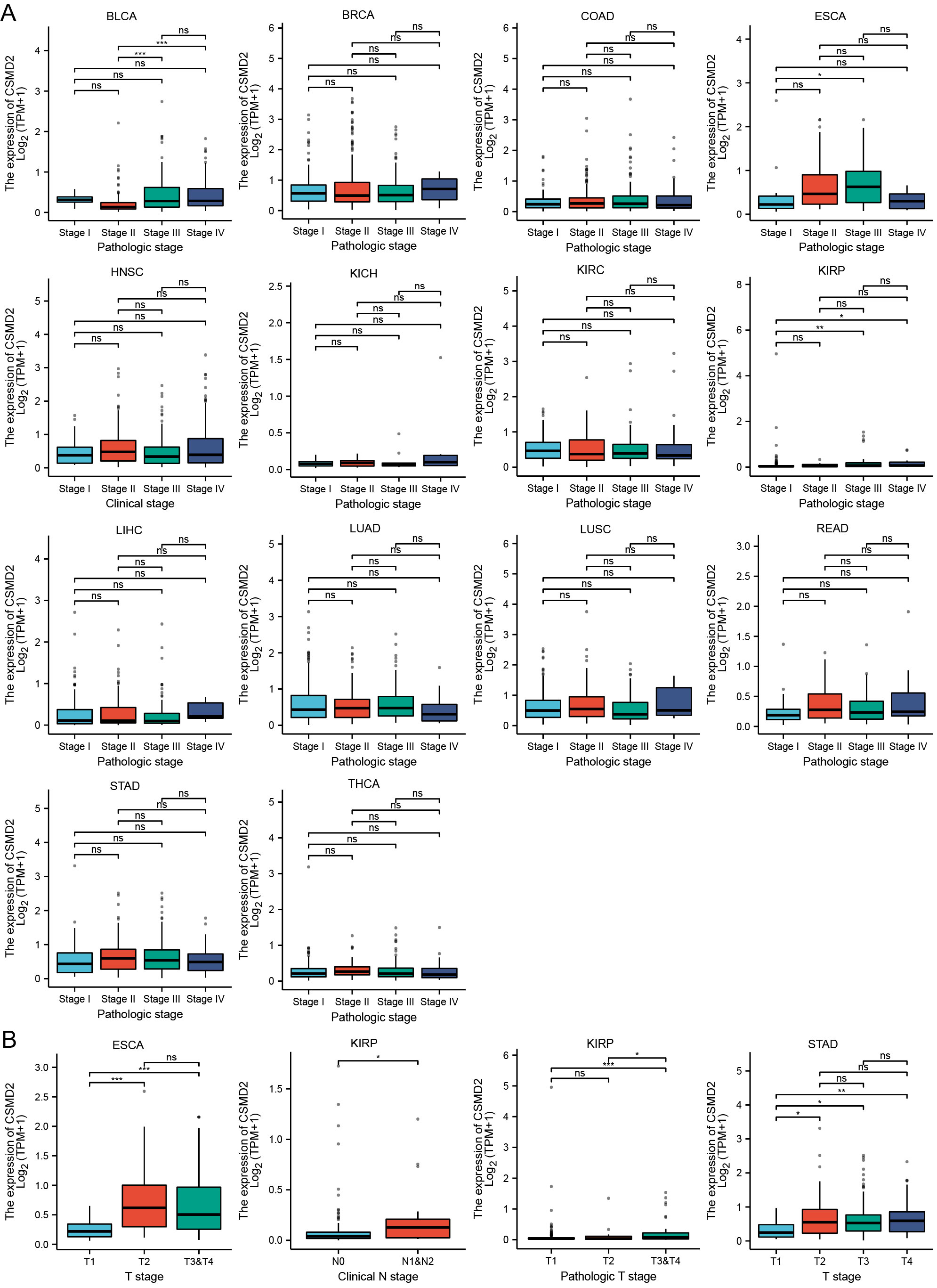

Supplement: Supplementary file 1 [file DataSheet1.zip › Supplementary materials-2022Aug5/Supplementary Figure S1. The association of clinical or pathological stage with CSMD2 expression.jpg]

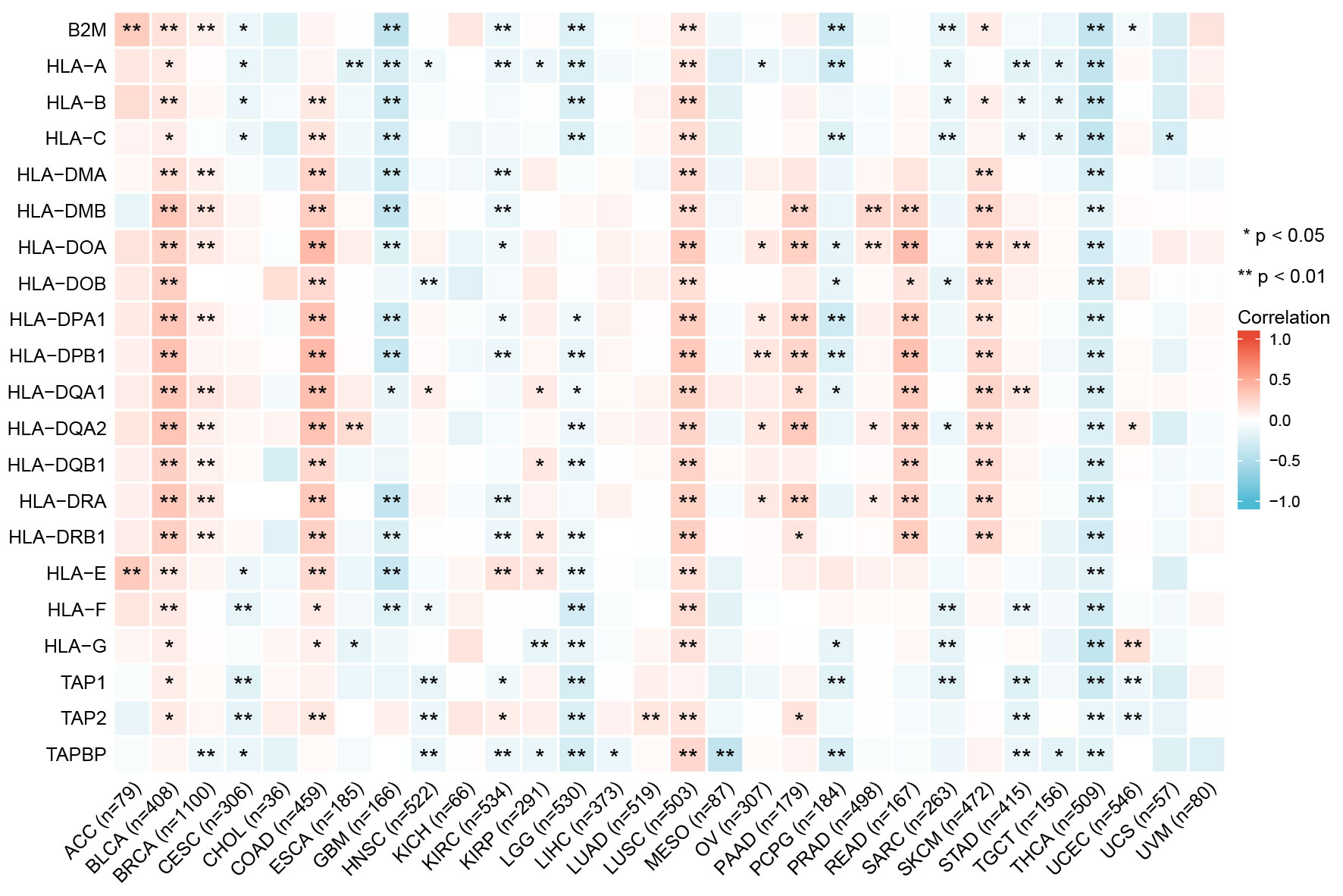

Supplement: Supplementary file 1 [file DataSheet1.zip › Supplementary materials-2022Aug5/Supplementary Figure S2. The correlation between CSMD2 and MHC molecule in tumors.jpg]
